# Classification of pediatric functional gastrointestinal disorders related to abdominal pain using Rome III vs. Rome IV criterions

**DOI:** 10.1186/s12876-018-0769-z

**Published:** 2018-03-17

**Authors:** Trent Edwards, Craig Friesen, Jennifer V. Schurman

**Affiliations:** 1 Division of Gastroenterology, Hepatology, and Nutrition, Children’s Mercy Kansas City, 2401 Gillham Road, Kansas City, MO 64108 USA; 2 Division of Developmental and Behavioral Sciences, Children’s Mercy Kansas City, 2401 Gillham Road, Kansas City, MO 64108 USA

**Keywords:** Abdominal pain, Functional dyspepsia, Irritable bowel syndrome, Rome criteria

## Abstract

**Background:**

The primary purpose of this study was to compare Rome III and IV evaluation criteria for irritable bowel syndrome (IBS), functional dyspepsia (FD), and an overlap syndrome consisting of both IBS and FD by assessing the frequency of each diagnosis in a population of children with chronic abdominal pain. Frequencies of Rome IV FD subtypes of postprandial distress syndrome (PDS) and epigastric pain syndrome (EPS) were determined and FD/IBS overlap symptom associations were also assessed.

**Methods:**

We conducted a cross-sectional retrospective chart review of 106 pediatric patients who had completed standardized medical histories as part of their evaluation for chronic abdominal pain. The patients ranged from eight to 17 years of age and reported having abdominal pain at least weekly for 8 weeks. Patients whose evaluation revealed gastrointestinal disease were excluded. The patients’ diagnoses were determined by a single pediatric gastroenterologist utilizing the specific criteria for Rome III and IV, respectively.

**Results:**

Patients were significantly more likely to be diagnosed with FD (84.9% vs. 52.8%), IBS (69.8% vs. 34%), and FD/IBS overlap (58.5% vs. 17.9%) by Rome IV criteria, as compared to Rome III criteria. With regard to Rome IV FD subtypes, 81.1% fulfilled criteria for PDS, 11.1% fulfilled criteria for EPS, 6.7% fulfilled criteria for both, and 1.1% did not fulfill criteria for either. Finally, we found an increased frequency of diarrhea and pain with eating in the overlap group compared to the non-overlap group of Rome III, while only an increased frequency of diarrhea was found in the overlap group compared to the non-overlap group of Rome IV.

**Conclusions:**

Our data demonstrate that utilizing Rome IV criteria, as compared to Rome III, results in an increase in the diagnosis of FD, a two-fold increase in the diagnosis of IBS, and a three-fold increase in the diagnosis of FD/IBS overlap. Rome IV criteria appears to result in greater heterogeneity within diagnostic categories. It is important to determine whether Rome IV diagnoses are predictive of treatment response, and if so, whether assessing symptom variability within a diagnosis will enhance the ability to select patients for a particular treatment.

## Background

Chronic or recurrent abdominal pain is common in children and adolescents, affecting up to 19% of children worldwide [1]. The majority of children/adolescents with chronic or recurrent abdominal pain will have symptoms that fit into a number of discrete diagnostic entities, broadly falling under the heading of functional gastrointestinal disorders (FGIDs) [2]. Criteria for these disorders were initially defined, and subsequently revised, multiple times by expert panels to create what are commonly known as Rome criteria. Under these criteria, there are four diagnoses related to abdominal pain, the most common being irritable bowel syndrome (IBS) and functional dyspepsia (FD) [3, 4]. Rome III criteria were released in 2006 and were subsequently revised to Rome IV criteria in 2016 [2, 5]. The IBS criteria for Rome III specified that abdominal pain had to be present with two or more of the following symptoms: improved with defecation, onset associated with a change in stool frequency, and onset associated with a change in stool form. The Rome IV criteria for IBS changed to become more inclusive so that it required only one of the previously listed symptoms, along with changing the criteria from pain improved with defecation to pain related to defecation (i.e., could improve or worsen) [5]. FD criteria also changed from Rome III to Rome IV, but with a focus on increasing specificity of diagnosis. Specifically, the Rome III criteria for FD utilized a broad description of upper abdominal pain or discomfort, while the Rome IV criteria for FD changed to require one or more specifically defined “bothersome” symptoms, including epigastric pain or burning, early satiety, and post-prandial bloating that further track into FD subtypes (i.e., post-prandial distress syndrome, epigastric pain syndrome) that may exist alone or overlap with one another [5].

Research shows that children can be classified as having both FD and IBS, and are not solely limited to one diagnosis in terms of naturally occurring symptom presentation [4, 6]. Previous studies evaluating IBS and FD have shown a large range of overlap with patients qualifying for both disorders simultaneously based on symptoms [7]. A recent study performed in Korea found that 110 out of 632 (17.4%) patients qualified as having both IBS and FD using Rome III criteria [8]. Given that Rome IV criteria are less stringent for the diagnosis of IBS and that FD may be diagnosed in the absence of upper abdominal pain if either “bothersome” early satiety, epigastric burning, or postprandial bloating are reported, it is certainly possible that the rate of a combined FD/IBS diagnosis would increase under the new criteria [5].

The primary aim of this study was to examine the evaluation criteria for IBS and FD in both Rome III and IV by assessing the frequency of each diagnosis in a population of children with chronic abdominal pain, as well as the overall impact of the criteria change on diagnosis rates for each condition. A second aim was to assess the frequencies of the Rome IV FD subcategories of postprandial distress syndrome (PDS) and epigastric pain syndrome (EPS). Our final aim was to compare the frequency of FD/IBS overlap when using Rome III versus Rome IV criteria. Understanding discrepancies between Rome III and Rome IV diagnoses is important in reconciling previous studies utilizing Rome III criteria with future studies utilizing Rome IV criteria.

## Methods

### Study design

This cross-sectional study utilized a retrospective chart review of pediatric patients who were diagnosed with FGIDs after being evaluated for chronic abdominal pain at Children’s Mercy Kansas City (CMKC) from October 12th, 2015 - June 20th, 2016. Standardized medical histories were analyzed. This study was approved by the CMKC institutional review board.

### Study cohort and exclusion criteria

Consecutive patients diagnosed with a FGID related to abdominal pain by a single pediatric gastroenterologist in an abdominal pain clinic were evaluated. The patients ranged from 8 to 17 years old and reported having abdominal pain at least once weekly for a minimum of 8 weeks. Patients whose evaluation revealed gastrointestinal disease were excluded. Seven patients were excluded from the study with excluding diagnoses consisting of celiac disease (2 patients), gastric erosions, gastric ulcer, duodenal ulcer, *H. Pylori*-associated nodular gastritis, and erosive esophagitis. Additionally, three patients originally diagnosed with Functional Abdominal Pain Syndrome by both Rome III and Rome IV criteria were excluded, as the focus of the current study was on movement within the diagnostic categories of IBS and FD. Patients having either mild histologic esophagitis (peak eosinophils < 5 per 400X field) or mild chronic gastritis were not excluded from the study cohort.

### Data sources

#### Gastrointestinal and systemic symptoms

All patients that fit the inclusion criteria had previously completed a standardized medical history form as part of the routine clinical evaluation for chronic abdominal pain. The standardized form included questions regarding the occurrence of abdominal pain, change in frequency or consistency of stools, and the presence of relief from defecation. Furthermore, patients labeled the location of their pain on a diagram of an abdomen that was placed on the form. Patients also indicated if the pain woke them up at night and if it increased following a meal. Further questions included whether the subjects experienced nausea or vomiting, early satiety, early satiety that prevented them from finishing a regular sized meal, and/or postprandial bloating. Other gastrointestinal symptoms assessed included having loose or hard stools, mucus or blood in stool, excessive flatulence, heart burn, acid regurgitation, diarrhea, or constipation.

Information acquired from this standardized form was entered into a database and used to classify their condition using the pediatric Rome III and Rome IV criteria, respectively. Under Rome III criteria, the patient was considered to have FD if they reported any upper abdominal pain not relieved by stooling and not associated with a change in stool frequency or form. Under Rome IV, a diagnosis of FD was made if the patient reported “bothersome” early satiety, postprandial bloating, or epigastric pain or burning unrelated to stool symptoms. FD was further classified per Rome IV as postprandial distress syndrome (PDS) if they had postprandial bloating or early satiety that prevented them from finishing a normal sized meal, and/or as epigastric pain syndrome (EPS) if they had pain or burning localized only to the epigastrium. IBS was diagnosed by Rome III if they had at least two of the following symptoms or Rome IV if they had at least one of the following symptoms: a change in stool frequency, a change in stool form, or if pain was relieved by a stool. As the questionnaire had been created, and information collected, before the Rome IV criteria were released, information regarding whether pain increased with a stool was not ascertained. When IBS classification did not match between Rome III and Rome IV (i.e., no IBS diagnosis under Rome III, but fulfilled IBS criteria under Rome IV), the symptom accounting for the new Rome IV diagnosis was recorded and enumerated. Diagnosis of an overlap syndrome consisted of having both FD and IBS within each respective Rome criterion.

### Statistical analysis

SPSS Version 23 (SPSS Inc, Chicago, IL) was used to perform statistical analysis. The frequencies of FD and IBS and FD/IBS overlap syndrome were compared between Rome III and Rome IV criteria using chi-square analysis or Fischer’s exact test as appropriate based on cell frequencies. Comparisons also were made between patients with FD/IBS overlap versus no overlap by Rome III and Rome IV criteria, respectively, on gender, age group (12 and under versus 13 and older), and the presence/absence of specific symptoms using chi-square analysis or Fischer’s exact test as appropriate. *P* values below 0.05 were considered statistically significant.

## Results

One hundred and six patients with a mean age of 13.4 years (range = 8–17 years) were evaluated. The majority (70.8%) of patients were female. Daily pain was reported by 68.9% of patients, pain several times per week by 21.7%, and weekly pain by 9.4%. The frequencies of specific gastrointestinal symptoms can be found in Table 1.

**Table 1:** Frequency of specific gastrointestinal symptoms in patients with FD and IBS

Symptom	Number of PatientsReporting Symptom (%)
Nausea	90 (84.9)
Pain worse with eating	73 (68.9)
Early satiety	71 (67.0)
Pain wakes them from sleep	66 (62.3)
Prevented from finishing a full-sized meal	65 (61.3)
Pain relief with a stool	51 (48.1)
Postprandial bloating	50 (47.2)
Acid regurgitation	47 (44.3)
Change in stool consistency	42 (39.6)
Heartburn	40 (37.7)
Diarrhea	32 (30.2)
Vomiting	30 (28.3)
Flatulence	30 (28.3)
Change in stool frequency	42 (39.6)
Constipation	21 (19.8)

**Fig. 1:**
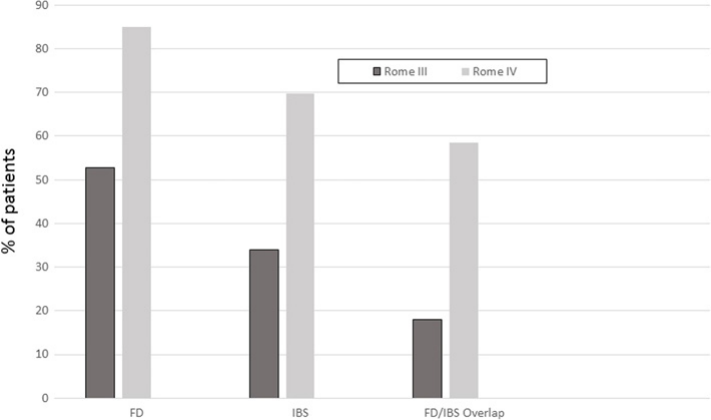
Frequency of diagnosis of functional dyspepsia (FD), irritable bowel syndrome (IBS), and FD/IBS overlap, respectively, utilizing Rome III vs. Rome IV criteria

Our primary aim was to examine the evaluation criteria for IBS and FD in both Rome III and IV by assessing the frequency of each diagnosis, as well as the overall impact of the criteria change on diagnosis rates for each condition. The diagnosis frequencies are shown in Fig. 1. As compared to Rome III criteria, utilizing Rome IV criteria resulted in a significant increase in the overall diagnosis of FD (84.9% vs. 52.8%; *p* < .05) and IBS (69.8% vs. 34.0%; *p* < .001), as well as the diagnosis of FD/IBS overlap specifically (58.5% vs. 17.9%; p < .001).

Given the difference in frequency of IBS between Rome III and IV, we further evaluated the cases where there were discrepant diagnoses. For a patient to be diagnosed with IBS by Rome IV but not Rome III, they would have to have one and only one of the three stool-related criteria. Therefore, we assessed what single stool related symptom was present. Results showed that a change in stool frequency was the symptom present in 2.6% (*n* = 1) of cases, a change in stool form was present in 34.2% (*n* = 13) of cases, and pain relief with stool was the symptom present in 63.2% (*n* = 24) of these discrepant cases.

Our second aim was to assess the frequencies of the Rome IV FD subcategories of postprandial distress syndrome (PDS) and epigastric pain syndrome (EPS). Of the 90 patients diagnosed with FD by Rome IV criteria, we found that 81.1% (*n* = 73) fulfilled criteria for PDS, 11.1% (*n* = 10) fulfilled criteria for EPS, 6.7% (*n* = 6) fulfilled criteria for both PDS and EPS, and 1.1% (*n* = 1) did not fulfill criteria for either PDS or EPS.

Finally, in an attempt to better understand the population of patients who were diagnosed with the overlap syndrome, we examined a variety of demographic/GI symptoms to see whether any of these would differ between the overlap and non-overlap groups in Rome III and IV, respectively. In order to avoid confounding the results, we chose to focus only on variables that were not part of the diagnostic criteria itself for FD and IBS in Rome III and IV. For patients with overlap diagnosed by Rome III criteria, diarrhea (55.6% in overlap, 25.2% in non-overlap; *p* < 0.05) and pain with eating (89.5% in overlap, 64.4% in non-overlap; *p* < 0.05) occurred more frequently in the overlap group than in IBS or FD alone; there also was a trend toward increased frequency of nausea (100% in overlap, 81.6% in non-overlap; *p* = 0.07) in the overlap group. For patients with overlap diagnosed by Rome IV criteria, diarrhea (41% in overlap, 15.9% in non-overlap; *p* = 0.006) was the only symptom to occur more frequently in the overlap group than in IBS or FD alone. There were no differences in overlap versus non-overlap patients with regard to age, sex, frequency of vomiting, or frequency of night waking with pain based on group assignment for either Rome III or Rome IV criteria.

## Discussion

The current study demonstrates that utilizing Rome IV criteria versus Rome III criteria results in a significant increase in the rate of diagnosis for both FD and IBS. This is not surprising, particularly with regard to IBS, where the new Rome IV criteria require only one symptom, as opposed to two under Rome III. Because we only asked patients whether pain was relieved with stooling, the IBS rate may actually be somewhat higher than we found with Rome IV criteria, as patients also would have been classified as having IBS if they reported that stooling increased pain. Application of Rome IV criteria resulted in a more than doubling of the rate of diagnosis of IBS. This represents a radical change in patient classification, which in turn requires that further work be done to determine which classification system better predicts prognosis or, more importantly, patient response to specific treatments. These findings also indicate that studies utilizing Rome III criteria cannot be pooled with studies utilizing Rome IV criteria. Instead, we will need to build a new knowledge base regarding FGIDs, as defined by Rome IV, in order to advance our understanding and efforts at defining treatment guidelines.

We further evaluated which symptom was present when the IBS diagnosis was discrepant between Rome III and Rome IV criteria. In nearly two-thirds of these cases, pain relief with a stool accounted for the new Rome IV IBS diagnosis. This is an interesting finding given that it is not clear from previous studies whether pain relief with a stool actually fits into the IBS symptom complex. For example, our own group has previously demonstrated low factor loading on IBS for this symptom [9]. Likewise, Caplan and colleagues demonstrated the validity of FD and IBS utilizing factor analysis, with the exception of relief with defecation [10].

Along with a greater frequency of diagnosis for FD and IBS in Rome IV comes an increase in the rate of FD/IBS overlap, with a greater than 3-fold increase as compared to Rome III. Significant overlap between FD and IBS has been previously reported in children; however, some investigators report no overlap, instead diagnosing only IBS when stool symptoms are present [3, 4]. Rome IV decreases some ambiguity, as 2 of the 3 FD symptoms are not defined by relationships with stools. However, this also contributes to the increased rate of an overlap diagnosis. The presence of a significant rate of FD/IBS overlap raises questions about whether FD and IBS are two distinct entities in real life patients or whether the two should be viewed on a sliding scale, representing a continuum between the two conditions [11]. Rome IV significantly increases the rate of overlap diagnoses and appears to make overlap even more heterogeneous, with increased symptom variation at the individual patient level.

Given the high rate of overlap under Rome IV, we further investigated whether there were differences between patients with FD/IBS overlap as compared to patients with only FD or IBS alone by gender or age; although this has been reported in adults, we found no differences along these lines in our study cohort [12]. We also evaluated the frequencies of other gastrointestinal symptoms not directly considered in the diagnosis of either condition. Overlap under Rome III criteria was associated with an increased rate of diarrhea and pain with eating, as well as a trend towards an increased rate of nausea. Overlap under Rome IV was associated with an increased rate of diarrhea only. Whether these associated symptoms are related to different pathophysiology is not clear, but they at least indicate that overlap may be associated with even greater symptom complexity and heterogeneity. Nausea frequently complicated abdominal pain in the current study, being present in 84.9% of patients. This is similar to previous reports demonstrating a high frequency of nausea with significant effects on clinical outcome [13].

The current study demonstrated that the great majority of FD patients met criteria for an FD subtype, as would be expected given that the subtype symptoms are the primary criteria for FD in general. PDS was much more frequent than EPS, with a frequency over 80%. Turco and colleagues previously reported PDS in 47%, EPS in 17%, and overlapping PDS and EPS in 36% of a pediatric cohort [14]. Higher rates of PDS as compared to EPS also have been reported in adult cohorts [15, 16]. In the current study, we also demonstrated overlap with only four patients meeting criteria for EPS alone (i.e., without corresponding PDS). While PDS (as compared to non-PDS) has demonstrated associations with mucosal inflammation, psychological functioning, and other gastrointestinal symptoms, further work is needed to determine whether PDS, EPS, PDS/EPS, and FD-NOS are associated with different pathophysiologic processes and treatment outcomes [17].

The main limitation of the current study is that it was performed utilizing data obtained from a group of patients who had been referred to a subspecialty abdominal pain clinic. Therefore, the findings may not be generalizable to children and adolescents who have not sought medical care or to those receiving their evaluation and treatment in a primary care setting.

## Conclusion

Rome IV significantly increased the rates of diagnosis for FD, IBS, and FD/IBS overlap. Notably, the rate of IBS diagnosis more than doubled, primarily due to patients endorsing the single item of decrease in pain with stooling. There was a corresponding 3-fold increase in the rate of an FD/IBS overlap diagnosis. In sum, application of the Rome IV criteria appears to result in greater heterogeneity within diagnostic categories. It is important, as we move forward, to understand the variability within diagnostic categories and the effect they have on treatment response. There is a need to determine whether Rome IV diagnoses themselves are predictive of treatment response and, further, whether assessing symptom variability within a diagnosis will enhance our ability to select patients for a particular treatment. Ultimately, we will need to find ways to reliably match patients to effective treatments; it remains to be determined whether this can be done at the level of diagnostic category, symptom profile, associated pathophysiology, and/or other individual-level factors.

## Abbreviations

EPS: Epigastric pain syndrome
FD: Functional dyspepsia
FGID: Functional gastrointestinal disorder
hpf: High power field
IBS: Irritable bowel syndrome
PDS: Postprandial distress syndrome
